# Evidence That the Hormonal Contraceptive Pill Is Associated With Cosmetic Habits

**DOI:** 10.3389/fpsyg.2018.01459

**Published:** 2018-08-23

**Authors:** Carlota Batres, Aurélie Porcheron, Gwenaël Kaminski, Sandra Courrèges, Frédérique Morizot, Richard Russell

**Affiliations:** ^1^Department of Psychology, Gettysburg College, Gettysburg, PA, United States; ^2^Department of Psychology, Franklin and Marshall College, Lancaster, PA, United States; ^3^CHANEL Fragrance & Beauty Research & Innovation, Pantin, France; ^4^Laboratoire de Psychologie et NeuroCognition, Université Pierre Mendès-France, Grenoble, France; ^5^CNRS (UMR 5263), Cognition, Langues, Langage, Ergonomie, Université de Toulouse, Toulouse, France; ^6^Institut Universitaire de France, Paris, France

**Keywords:** cosmetics, makeup, contraception, birth control, grooming behaviors

## Abstract

Hormonal contraception is known to cause subtle but widespread behavioral changes. Here, we investigated whether changes in cosmetic habits are associated with use of the hormonal contraceptive pill. We photographed a sample of women (*N* = 36) who self-reported whether or not they use the contraceptive pill, as well as their cosmetic habits. A separate sample of participants (*N* = 143) rated how much makeup these target women appeared to be wearing. We found that women not using the contraceptive pill (i.e., naturally cycling women) reported spending more time applying cosmetics for an outing than did women who use the contraceptive pill. We also found that the faces of these naturally cycling women were rated as wearing more cosmetics than the faces of the women using the contraceptive pill. Thus, we found clear associations between contraceptive pill use and makeup use. This provides evidence consistent with the possibility that cosmetic habits, and grooming behaviors more generally, are affected by hormonal contraception.

## Introduction

The hormonal contraceptive pill is used by approximately 100 million women worldwide (Christin-Maitre, 2013). Its main function is to change the hormonal state of the menstrual cycle in order to mimic, and thus prevent, pregnancy (Alvergne and Lummaa, 2010). While the majority of women take the contraceptive pill to prevent pregnancy, approximately 14% of women use it for other reasons, such as for lessening menstrual pain and migraines (Cooper and Adigun, 2017).

In addition to its medical side effects, the contraceptive pill has also been linked to several behavioral effects (Welling et al., 2012). For example, one study found that married women not using the contraceptive pill (i.e., naturally cycling women) showed an increase in female-initiated sexual behavior at the time of ovulation, whereas women using the contraceptive pill did not show such a rise (Adams et al., 1978). Other studies have also found that the hormonal state of the contraceptive pill changes women’s partner preferences. For instance, unlike naturally cycling women, one study found that those using the contraceptive pill prefer less masculine male faces (Little et al., 2002, but see Jones et al., 2018).

Research has also found that the contraceptive pill affects women’s ability to attract mates. For example, one study found that for naturally cycling women, their voices became more attractive as their risk of conception increased but no such effect was found for women using the contraceptive pill (Pipitone and Gallup, 2008). One field study examined the tip earnings of professional lap dancers and found that those who were naturally cycling made more money per shift than those who were using the contraceptive pill (Miller et al., 2007).

In this study, we aimed to examine whether the use of the contraceptive pill also influences grooming behaviors, particularly cosmetic habits. Previous research has found that among naturally cycling women, the time spent putting on cosmetics, and the rated level of cosmetics used by women, seems to be higher near ovulation (Guéguen, 2012). No study, however, has examined whether there is a difference in cosmetic habits between naturally cycling women and women using the contraceptive pill. Previous research has shown that, unlike women using the contraceptive pill, naturally cycling women change their appearance near ovulation to look more attractive (Haselton et al., 2007; Durante et al., 2011). Given that makeup is one way that women can alter their attractiveness (Graham and Jouhar, 1981; Jones et al., 2015; Jones and Kramer, 2016; Batres et al., 2018), we predicted that naturally cycling women would report spending more time applying cosmetics, and would be rated as wearing more cosmetics, than women using the contraceptive pill.

## Study 1

### Methods

#### Participants and Procedure

Thirty six women (*M* age = 19.58 years, *SD* = 2.14) completed the study. The participants were recruited at the University of Grenoble using advertisements and obtained financial compensation to participate in the study. The research was performed in accordance with the Declaration of Helsinki: it was conducted with the understanding and the written consent of each participant (who were instructed that their photographs would be taken) and was approved by local ethics boards (CNRS and the University of Grenoble).

After arriving at the laboratory, 20 min were allowed to pass in order to allow the participants’ skin to acclimatize to the indoor temperature. Photographs were then taken of each participant facing forward, under constant camera/lighting conditions, with neutral expressions, and closed mouths. Each participant also completed a questionnaire in which she reported whether she was taking hormonal contraceptives, if so, what type of hormonal contraceptives (e.g., the contraceptive pill, an intrauterine device), whether she had regular menstrual cycles, and whether she was in a relationship. All 36 women reported having regular menstrual cycles. Twenty women reported not using any hormonal contraceptives (i.e., naturally cycling) and 16 women reported using hormonal contraceptives, out of which 100% reported using the contraceptive pill.

Each participant also completed two questions pertaining to her cosmetic habits: how much time she spent on her daily makeup in the morning and how much time she spent making up for an outing. These two questions allowed us to define two variables concerning cosmetic habits: daily cosmetics and outing cosmetics. To examine the association between contraceptive pill use and cosmetic habits, we performed independent samples *t*-tests on both cosmetic habits variables.

### Results

**FIGURE 1:**
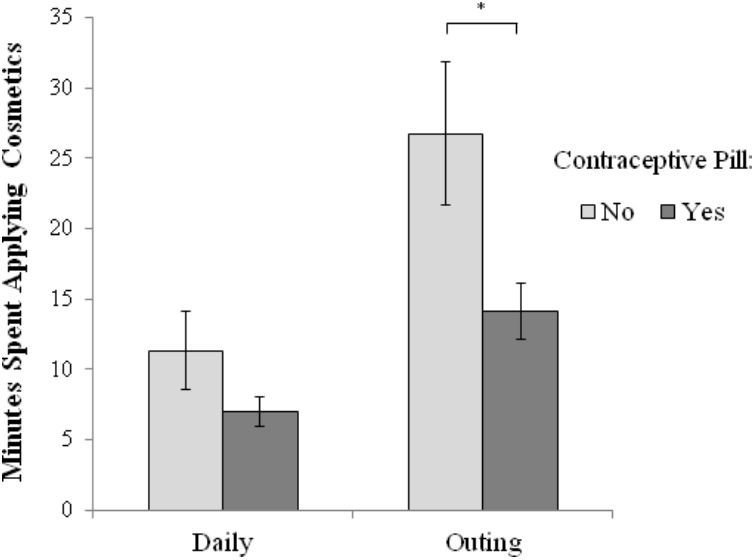
In minutes, reported amount of time spent applying daily cosmetics in the morning and reported amount of time spent making up for an outing, for both naturally cycling women (i.e., those not using the contraceptive pill) and women using the hormonal contraceptive pill. The asterisk indicates a significant difference (^∗^*p* < 0.05). Error bars indicate the standard error of the mean.

Naturally cycling women reported spending more time applying cosmetics (both daily and for an outing) than women using the contraceptive pill (see **Figure 1**). The difference between naturally cycling women and women using the contraceptive pill was not statistically significant for the amount of time they spent on their daily cosmetics, *t*(33) = 1.59, *p* = 0.121, Cohen’s *d* = 0.51, but it was statistically significant for the amount of time they spent on their cosmetics for an outing, *t*(19.6) = 2.30, *p* = 0.033, Cohen’s *d* = 0.80.

## Study 2

### Methods

#### Participants and Procedure

One hundred and forty three Gettysburg College students participated in Study 2 (*M* age = 18.53 years, *SD* = 0.87, 56 male, 87 female) as part of a course requirement. Ethical approval was received from the Gettysburg College Institutional Review Board. Participants were instructed that they would be viewing and rating face images on a computer. Participants were asked to rate each face on the question: “How much makeup does this face have?,” where 1 = Very little makeup and 7 = A lot of makeup. The faces presented were those of the women from Study 1. To examine the association between pill use and perceived amount of cosmetics, we performed a linear mixed model.

### Results

**FIGURE 2:**
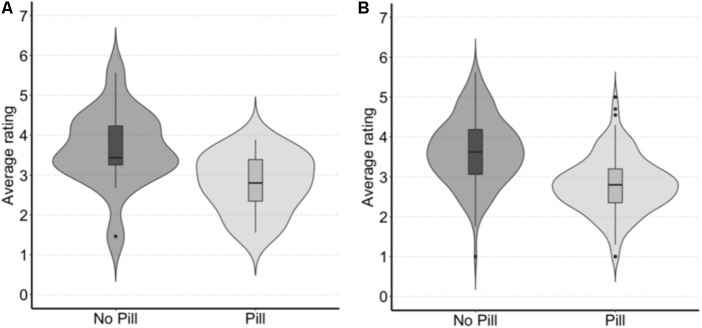
Average rated amount of cosmetics (where 1 = Very little makeup and 7 = A lot of makeup) by **(A)** target faces and **(B)** participants, for both naturally cycling women (i.e., those not using the contraceptive pill) and women using the contraceptive pill.

Due to repeated measurements, we included the target faces and the participants as random effects. Naturally cycling women were rated as having higher levels of cosmetics [3.65 (3.24–4.06)] than women using the contraceptive pill [2.79 (2.43–3.17)] (see **Figure 2**). We found that the difference in amount of perceived cosmetics between naturally cycling women and women using the contraceptive pill was statistically significant [χ2(1) = 8.86, *p* = 0.003, β = 0.85 [0.31–1.39)].

## Discussion

The results from Studies 1 and 2 provide the first evidence that the use of the hormonal contraceptive pill is associated with cosmetic habits. In Study 1, we found that naturally cycling women spent more time applying cosmetics for an outing than women using the contraceptive pill. On average, naturally cycling women reported spending an extra 13 min applying cosmetics for an outing than women using the contraceptive pill. Naturally cycling women also reported spending more time applying their daily cosmetics than women using the contraceptive pill, but this difference was not statistically significant. In Study 2, we found that the faces of the naturally cycling women (taken during the day) were rated by participants as having higher amounts of cosmetics than the faces of the women using the contraceptive pill. This suggests that while naturally cycling women may not spend more time applying their daily makeup, they use more visible cosmetics. These findings thus provide evidence that contraceptive pill use is associated with cosmetics use.

This is consistent with previous research suggesting that naturally cycling women are found more attractive than women using the hormonal contraceptive pill (Miller et al., 2007). Our study, however, proposes that part of such difference in attractiveness may be due to cosmetics. In other words, naturally cycling women may, in part, be found more attractive than women using the contraceptive pill because they wear more cosmetics, which greatly increases attractiveness (Graham and Jouhar, 1981; Batres et al., 2018). For instance, Miller et al.’s (2007) study found that naturally cycling lap dancers had higher tip earnings than those using the contraceptive pill, but part of that difference may be explained by a change in the amount of cosmetics worn by the dancers. Indeed, research has observed that waitresses receive higher tips when they are wearing cosmetics compared to when they are not (Jacob et al., 2009). This suggests it is likely that different makeup use explains part of the differences in attractiveness found between naturally cycling women and women using hormonal contraception.

Our results may also point to a larger issue: that the contraceptive pill may be associated with behavioral changes that affect women’s grooming practices more generally, of which cosmetics is just one part. In other words, the contraceptive pill may suppress how women adorn themselves, otherwise referred to as the extended phenotype (Etcoff et al., 2011). Some studies have examined how the extended phenotype changes throughout the menstrual cycle. For instance, one study found that self-grooming and ornamentation through attractive choice of dress increased during high fertility periods (Haselton et al., 2007). However, a recent longitudinal study did not find evidence for fertility-linked changes in women’s clothing choices (Arslan et al., 2017). Similar studies are needed to compare the extended phenotype of women who are using the contraceptive pill and those that are not. Such research would shed light on whether cosmetic habits are the only form of ornamentation influenced by the contraceptive pill or whether it is just one facet of a greater behavioral shift in how women present themselves (e.g., grooming, jewelry, clothing).

Our study did not control the phase of the menstrual cycle in which women were photographed nor asked how long the women had been using or not using the contraceptive pill and therefore future research should control for this. We also did not define what was meant by an outing and the interpretation of this could have had some impact on self-reported time spent applying cosmetics. For example, women may have reported less time if an outing was interpreted as going out for dinner versus if it was interpreted as going out to a club. Future studies would therefore benefit from being more specific when asking about time spent applying cosmetics for an outing. Similarly, while we asked the women how much time they spend on their daily makeup in the morning, it would have been helpful to ask how much time they spent making up that exact morning in order to better link cosmetics application time with perceptual differences. Moreover, adding other measures of cosmetics use would also be beneficial in future studies (e.g., “How natural does the makeup on this face look?”). Women who are skilled at perfecting the “natural” look are likely to spend more time applying cosmetics but may appear to be wearing less makeup.

Further research is still needed to better understand links between the contraceptive pill and cosmetic habits as our study was a non-randomized between-subjects design and research has found that naturally cycling women and women using the contraceptive pill differ in other ways (Alexander et al., 1990). For example, Little et al. (2002) found that women using the contraceptive pill reported having more lifetime sexual partners than naturally cycling women. A related concern is that in our study only 19% of naturally cycling women reported being in a relationship, while 85% of women using the contraceptive pill reported being in a relationship. Our sample was too small to include relationship status as a factor in our analyses and thus future research with a larger sample is needed. No study has examined differences in cosmetic habits between single women and women in a relationship, however, such a difference could very well be possible. In order to address these two concerns and confirm the link between the contraceptive pill and cosmetic habits, an experimental design, rather than the correlational one used here, would be needed. More specifically, women would need to be randomly assigned to use or not use the contraceptive pill in order to be able to establish a casual effect between the contraceptive pill and cosmetic habits. However, such an experiment is unlikely due to ethical reasons, which is why the current literature has relied on non-randomized between-subjects designs (e.g., Adams et al., 1978; Little et al., 2002; Miller et al., 2007; Pipitone and Gallup, 2008; Welling et al., 2012).

## Conclusion

In conclusion, we found that, compared to women using the contraceptive pill, naturally cycling women self-reported spending more time applying cosmetics for an outing, and their faces were rated as having higher levels of cosmetics. These results provide initial evidence that the contraceptive pill is associated with cosmetic habits. Moreover, this association may be part of a broader relationship between contraceptive pill use and other grooming behaviors, stemming from hormone-mediated changes in motivations.

## Ethics Statement

The experiments were undertaken with the understanding and written consent of each subject, with the approval of the appropriate local ethics committees, and in compliance with national legislation and the Code of Ethical Principles for Medical Research Involving Human Subjects of the World Medical Association (Declaration of Helsinki).

## Author Contributions

CB, AP, GK, FM, and RR conceived and designed the research. CB and GK acquired the data. CB, GK, and RR analyzed the data. CB, AP, GK, SC, and RR interpreted the results. CB drafted the work. CB, AP, GK, SC, and RR critically revised the paper. CB, AP, GK, SC, FM, and RR approved the final version to be published and agreed to be accountable for the content of the work.

## Conflict of Interest Statement

AP, FM, and SC work at CHANEL Fragrance & Beauty Research & Innovation, a cosmetics company, and CB and RR receive funding from CHANEL Fragrance & Beauty Research & Innovation. The remaining author declares that the research was conducted in the absence of any commercial or financial relationships that could be construed as a potential conflict of interest.
